# Association of International Editorial Staff With Published Articles From Low- and Middle-Income Countries

**DOI:** 10.1001/jamanetworkopen.2022.13269

**Published:** 2022-05-23

**Authors:** Gandolina Melhem, Chris A. Rees, Bruno F. Sunguya, Mohsin Ali, Anura Kurpad, Christopher P. Duggan

**Affiliations:** 1 Precision Vaccines Program, Boston Children’s Hospital, Boston, Massachusetts; 2 Division of Pediatric Emergency Medicine, Emory University School of Medicine, Atlanta, Georgia; 3 Emergency Medicine, Children’s Healthcare of Atlanta, Atlanta, Georgia; 4 School of Public Health and Social Sciences, Muhimbili University of Health and Allied Sciences, Dar es Salaam, Tanzania; 5 Division of Infectious Diseases, Department of Paediatrics, The Hospital for Sick Children, Toronto, Ontario, Canada; 6 Department of Physiology and Nutrition, St John’s Medical College, Bengaluru, India; 7 Center for Nutrition, Division of Gastroenterology, Hepatology and Nutrition, Boston Children’s Hospital, Boston, Massachusetts; 8 Department of Nutrition, Harvard T.H. Chan School of Public Health, Boston, Massachusetts

## Abstract

**Importance:**

The association between geographic diversity of medical journal editorial staff and publications reporting research conducted in low- and middle-income countries (LMICs) is unclear.

**Objective:**

To examine the association between having editorial staff members affiliated with LMICs and publishing research articles from LMICs in leading biomedical journals.

**Design, Setting, and Participants:**

This cross-sectional study included biomedical journals in fields representing the largest disease burden globally from January 1 to December 31, 2020. Websites of the 5 leading journals in general medicine, pediatrics, surgery, obstetrics and gynecology, cancer, cardiovascular diseases, infectious diseases, psychiatry, and nutrition were reviewed to obtain the country affiliations of editorial staff members. To determine article study countries, original research articles in each journal were reviewed through MEDLINE. Editorial staff country affiliations and study country locations were classified according to World Bank income brackets and regions.

**Exposure:**

Editorial staff country affiliation.

**Main Outcomes and Measures:**

Descriptive statistics of the proportion of editorial staff affiliated with each income bracket and region and Spearman rank correlation coefficients were used to assess the association between the proportion of editorial staff affiliated with LMICs and the proportion of published articles reporting work conducted in these countries.

**Results:**

There were 3819 editorial staff members in the 45 included journals: 3637 (95.2%) were affiliated with high-income countries, 140 (3.7%) with upper-middle-income countries, 37 (1.0%) with lower-middle-income countries, and 5 (0.1%) with low-income countries. All 48 editors-in-chief were affiliated with a high-income country. Editorial staff members were mostly affiliated with North American countries (n = 2120 [55.5%]) and European or Central Asian countries (n = 1256 [32.9%]). Of the 10 096 original research articles included in our analysis, 7857 (77.8%) reported research conducted in high-income countries, 1562 (15.5%) reported research conducted in upper-middle-income countries, 507 (5.0%) reported research conducted in lower-middle-income countries, and 170 (1.7%) reported research conducted in low-income countries. Greater editorial staff representation correlated moderately with more published articles reporting research conducted in LMICs (Spearman ρ = 0.51; 95% CI, 0.25-0.70; *P* < .001).

**Conclusions and Relevance:**

In this cross-sectional study, editorial staff in leading biomedical journals were largely composed of individuals affiliated with high-income countries in North America and Europe. A correlation was found between greater editorial staff representation and publication of research focused on LMICs, suggesting that the inclusion of editorial staff affiliated with LMICs may promote the publication of research conducted in those countries.

## Key Points

**Question:**

Do medical journals whose editorial staff includes more editors affiliated with low- and middle-income countries (LMICs) publish more original research conducted in LMICs?

**Findings:**

In this cross-sectional study, the inclusion of editorial staff affiliated with LMICs was moderately associated with a higher proportion of published articles reporting research conducted in LMICs.

**Meaning:**

This study suggests that inclusion of editorial staff affiliated with LMICs may be associated with the increased publication of work conducted in LMICs.

## Teaser

This cross-sectional study examines the association of having editorial staff members affiliated with low- and middle-income countries with publications reporting research from low- and middle-income countries in leading biomedical journals.

## Introduction

Most of the world’s infectious and chronic disease burden lies in low- and middle-income countries (LMICs),^1^ owing, in part, to socioeconomic differences contributing to high rates of undernutrition, unsafe water, poor sanitation and hygiene, and disparities in mortality and overall life expectancy.^1,2,3^ Despite LMICs having a significant share of the disease burden, several studies have reported major deficits in research originating from, and addressing, diseases common in those countries.^4,5,6,7^ The current scientific paradigm is such that research is disproportionately centralized in wealthier nations of the so-called Global North, resulting in some dependence among LMICs on “trickle-down science” from high-income countries (HICs).^8^

In addition to the scarcity of original research emerging from LMICs, individuals from LMICs are also underrepresented as editorial staff members of major medical journals.^9,10,11,12^ One study revealed that 68% of editorial staff members of leading global health journals are based in HICs and that, with few exceptions, the editors-in-chief of these journals are men and are based in high-income European and North American countries.^10^ Furthermore, some scientists based in LMICs report perceived biases among editorial staff and systemic barriers that inhibit engagement with the greater scientific community and subsequent scientific publication.^13,14^

Despite the well-documented lack of representation of individuals from LMICs in editorial staff, it is unclear whether greater geographic diversity among editorial staff is associated with more publications reporting research conducted in LMICs. Such an understanding may elucidate a potential way to foster a geographically diverse representation of published work that addresses problems in countries where the disease burden is greatest. Our objective was to assess the association between the affiliated countries of editorial staff members and the income bracket of study countries for articles published in leading journals. We hypothesized that greater representation from LMICs among editorial staff would be associated with more published articles from LMICs. We postulated this based on the assumption that editorial staff members from LMICs may place greater value on, and understand the relevance of, research conducted in settings similar to their own.

## Methods

### Study Design

We conducted a cross-sectional study including leading biomedical journals in fields representing the largest disease burden globally from January 1 to December 31, 2020. Because we used publicly available data from journal websites and articles published in peer-reviewed journals, this study was exempted from review by the institutional review board at Boston Children’s Hospital. Informed consent was not required because all data were obtained from journal websites and published articles. This study followed the Strengthening the Reporting of Observational Studies in Epidemiology (STROBE) reporting guideline.

### Data Sources

We reviewed the websites of the top 5 journals in the fields of general medicine, pediatrics, surgery, obstetrics and gynecology, cancer, cardiovascular diseases, infectious diseases, psychiatry, and nutrition (eAppendix in the Supplement). The top 5 journals in each category were ranked according to impact factor in the Web of Science Journal Citation Report in 2019.^15^ These disease categories correspond to leading causes of disability-adjusted life-years among all age groups in low-, low-middle, middle, and high-middle sociodemographic index countries according to the Institute for Health Metrics and Evaluation in 2019.^16^ Because we aimed to describe the publication rates of articles reporting primary research conducted in LMICs, we excluded review journals (eg, *Nature Reviews Clinical Oncology* and *Human Reproduction Update*). We also excluded journals that were not clinically focused (eg, *Food Chemistry*), given the greater likelihood of such journals publishing primarily laboratory-based studies. We reviewed articles published in each selected journal from January 1 to December 31, 2020, using MEDLINE through the search engine PubMed.

### Data Extraction

We reviewed journal websites to obtain the country affiliations of staff members and their editorial positions (eg, editor-in-chief, associate editor, and senior editor). We defined *editorial staff* as including any individual listed as an editor in any capacity. We did not include ancillary journal staff, such as editorial managers. We recorded the names of the editorial staff, their editorial position, and their country affiliation in December 2020. For journals that did not include a country affiliation for editorial staff, we conducted a Google and PubMed search using the names of editorial staff members and topic areas related to the journal’s content to obtain their country affiliation. We queried PubMed for each selected journal by name to obtain the total number of published articles in 2020. We extracted the metadata (ie, article name, journal, and abstract) of each article through EndNote, version 20 (Clarivate).^17^

### Exclusion Criteria

To target primary research articles, we excluded articles that had no abstract or no listed authors because these articles were likely to be nonoriginal research articles. Thereafter, 2 of us (G.M. and C.A.R.) reviewed all of the remaining article titles and abstracts to exclude nonoriginal research articles. We excluded editorial articles, letters to the editor, historical articles, case reports, commentaries, viewpoints, case series, clinical practice guidelines, biographies, conference summaries, clinical trial protocols, interviews, and review articles (including systematic reviews and meta-analyses) because these articles were less likely to report study countries.

### Editorial Staff Country Assignment

We extracted editorial staff members’ country affiliations as listed on the journals’ websites and assigned these countries an income bracket (eg, HICs, upper-middle-income countries, lower-middle-income countries, and low-income countries) and a region (eg, East Asia and Pacific, Europe and Central Asia, Latin America and the Caribbean, Middle East and North Africa, North America, South Asia, and sub-Saharan Africa) according to World Bank classifications.^18^

### Study Country Assignment

We searched for each country name in the article title, abstract, key word, and medical subject heading (MeSH) fields using EndNote to determine in which country, or countries, a study was conducted. Studies conducted in more than 1 country were assigned to each country in which the study was conducted. We randomly selected a subset of 20% of articles assigned a study country through this approach to validate the study country assignment process. For articles published in the selected journals but not assigned a study country through this approach, 2 of us (G.M. and C.A.R.) reviewed each article, including the full text, to assign a study country. In rare cases in which no study country was explicitly stated in the article text, study country was assigned according to the country of all of the authors’ affiliations. Study countries for each original research article were assigned an income bracket and a region according to the World Bank classifications.^18^

### Statistical Analysis

We calculated descriptive statistics of editorial staff by country income bracket and geographic region, as well as the number of articles reporting work conducted in each income bracket and geographic region. To assess the association between editorial staff affiliated with LMICs and publications from LMICs, we created scatterplots and calculated Spearman ρ coefficients and 95% CIs to assess the correlation between the proportion of each journal’s editorial staff affiliated with an LMIC and the proportion of articles reporting work conducted in LMICs. All *P* values were from 2-sided tests and results were deemed statistically significant at *P* < .05. Because we hypothesized that multicountry studies would be published in leading journals regardless of the affiliations of the editorial staff, we conducted additional subanalyses among multicountry and single-country studies. All analyses were conducted using R, version 4.0.3 (R Group for Statistical Computing).

## Results

### Characteristics of Editorial Staff

There were 45 journals included in our analysis (5 for each of the 9 disease fields representing the largest disease burden globally). All included journals had main offices based in North America or Europe, with the exception of *The Lancet*, which has offices in the United States, United Kingdom, and China.

In 2020, there were 3819 editorial staff members in the included journals. Of those, 3637 (95.2%) were affiliated with HICs, 140 (3.7%) with upper-middle-income countries, 37 (1.0%) with lower-middle-income countries, and 5 (0.1%) with low-income countries (Table 1). All 48 editors-in-chief were affiliated with an HIC. Of the 459 associate editors, 445 (96.9%) were affiliated with HICs, 10 (2.2%) were affiliated with upper-middle-income countries, 4 (0.9%) were affiliated with lower-middle-income countries, and no associate editors were affiliated with low-income countries.

**Table 1.** Editorial Staff Affiliations in Leading Medical Journals by Country Income Category

Journal topic	Total staff, No.	No. (%) of editorial staff members			
		High	Upper-middle	Lower-middle	Low
General medicine	517	506 (97.9)	8 (1.5)	1 (0.2)	2 (0.4)
Surgery	411	395 (96.1)	14 (3.4)	2 (0.5)	0
Pediatrics	156	155 (99.4)	1 (0.6)	0	0
Obstetrics and gynecology	385	371 (96.4)	11 (2.9)	3 (0.8)	0
Cancer	207	199 (96.1)	5 (2.4)	3 (1.4)	0
Cardiovascular diseases	1022	968 (94.7)	48 (4.7)	6 (0.6)	0
Infectious diseases	429	396 (92.3)	23 (5.4)	7 (1.6)	3 (0.7)
Psychiatry	192	169 (88.0)	13 (6.8)	10 (5.2)	0
Nutrition	500	478 (95.6)	17 (3.4)	5 (1.0)	0
Total	3819	3637 (95.2)	140 (3.7)	37 (1.0)	5 (0.1)

Of the 140 editorial staff members affiliated with upper-middle-income countries, 29 (20.7%) were advisory board members, and 30 (21.4%) were listed as editorial board members with no further description. Of the 37 editorial staff members affiliated with lower-middle-income countries, 16 (43.2%) were advisory board members, and 9 (24.3%) were editorial board members. All 5 of the editorial staff members affiliated with low-income countries were listed as international advisory members.

In addition, most editorial staff members in the included journals were affiliated with North American (n = 2120 [55.5%]) and European and Central Asian countries (n = 1256 [32.9%]) (eTable 1 in the Supplement). Regions such as sub-Saharan Africa (n = 27 [0.7%]), South Asia (n = 24 [0.6%]), and Latin America and the Caribbean (n = 55 [1.4%]) had little editorial staff representation.

### Characteristics of Published Original Research Articles

**Figure 1.**
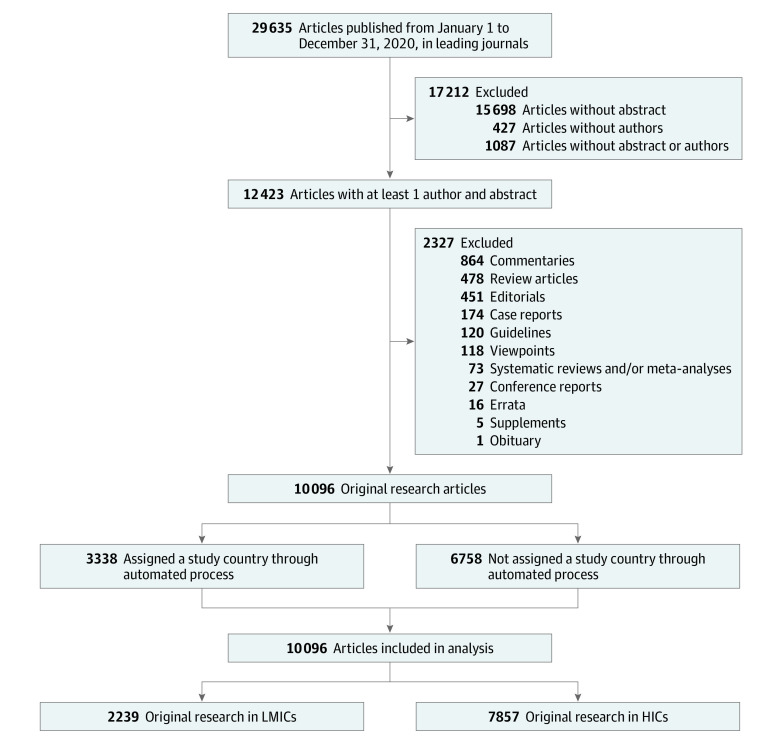
Inclusion of Articles in Analysis of Editorial Staff and Original Research Study Country HICs indicates high-income countries; LMICs, low- and middle-income countries.

A total of 29 635 articles were published in the included journals in 2020, of which 10 096 met inclusion criteria (Figure 1). There were 6758 articles that were assigned a study country through review by 2 of us (G.M. and C.A.R.), and 3338 were assigned a study country through the automated algorithm. The accuracy of the automated algorithm was 96.7% in correctly assigning the study country as identified through additional review of 667 articles (20.0%).

Of the 10 096 included articles, 7857 (77.8%) reported research conducted in HICs, 1562 (15.5%) reported research conducted in upper-middle-income countries, 507 (5.0%) reported research conducted in lower-middle-income countries, and 170 (1.7%) reported research conducted in low-income countries (Table 2). As in the geographic trends in editorial staff, published original research articles in the selected journals disproportionately originated from North America (n = 4088 [40.5%]) and Europe (n = 3344 [33.1%]) (eTable 2 in the Supplement). Research conducted in Latin America and the Caribbean (n = 500 [5.0%]), sub-Saharan Africa (n = 404 [4.0%]), South Asia (n = 249 [2.5%]), and the Middle East and North Africa (n = 322 [3.2%]) constituted a small proportion of original research articles published in the included journals (eTable 2 in the Supplement).

**Table 2.** Study Country Income Category of Original Research Articles Published in Leading Medical Journals

Journal topic	Total articles, No.^a^	No. (%) of articles			
		High	Upper-middle	Lower-middle	Low
General medicine	1070	790 (73.8)	192 (17.9)	71 (6.6)	17 (1.6)
Surgery	1189	1085 (91.3)	85 (7.1)	16 (1.3)	3 (0.3)
Pediatrics	779	715 (91.8)	32 (4.1)	24 (3.1)	8 (1.0)
Obstetrics and gynecology	1265	1049 (82.9)	182 (14.4)	30 (2.4)	4 (0.3)
Cancer	690	571 (82.8)	100 (14.5)	16 (2.3)	3 (0.4)
Cardiovascular diseases	1146	921 (80.4)	164 (14.3)	59 (5.1)	2 (0.2)
Infectious diseases	2149	1347 (62.7)	456 (21.2)	228 (10.6)	118 (5.5)
Psychiatry	243	218 (89.7)	20 (8.2)	5 (2.1)	0
Nutrition	1565	1161 (74.2)	331 (21.2)	58 (3.7)	15 (1.0)
Total	10 096	7857 (77.8)	1562 (15.5)	507 (5.0)	170 (1.7)

^a^ There were 309 articles that reported multicountry work conducted in countries with more than 1 income bracket.

### Association Between Editorial Staff Country Income Bracket and Study Countries

**Figure 2.**
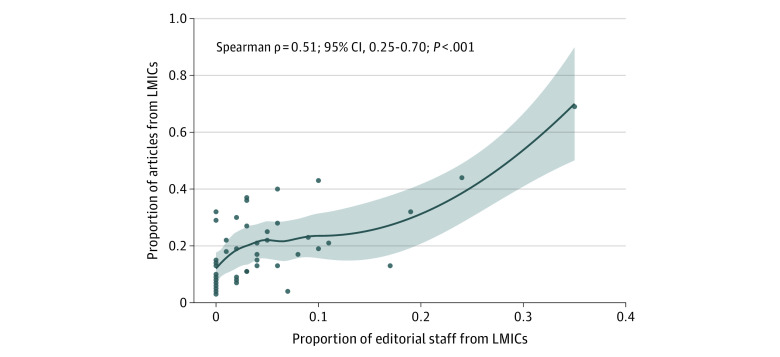
Association Between Affiliations of Editorial Staff Members and Publications From Low- and Middle-Income Countries (LMICs) The shaded area indicates the 95% CI.

The correlation between editorial staff country income bracket and original research articles from LMICs was moderate among all articles (Spearman ρ = 0.51; 95% CI, 0.25-0.70; *P* < .001) (Figure 2). When limited to studies conducted in multiple countries, the correlation was lower (Spearman ρ = 0.42; 95% CI, 0.14-0.63; *P* = .005) (eFigure 1 in the Supplement). Among single-country studies, the Spearman ρ was 0.47 (95% CI, 0.20-0.67; *P* = .001) (eFigure 2 in the Supplement). The correlation between editorial staff geographic region and the proportion of publications reporting work conducted in each geographic region varied by region (eFigure 3 in the Supplement). Among journals that published more than 100 articles, there was moderate correlation between editorial staff affiliation with LMICs and articles reporting work conducted in LMICs (Spearman ρ = 0.54; 95% CI, 0.25-0.74; *P* < .001).

## Discussion

In our analysis including 45 leading journals, 3819 editorial staff members, and 10 096 original research articles, editorial staff members affiliated with LMICs were underrepresented compared with editorial staff members affiliated with HICs. When editorial staff members affiliated with LMICs were included, they were seldom found in senior roles, such as editor-in-chief or associate editor. The inclusion of more editorial staff members affiliated with LMICs was moderately correlated with more publications of original research conducted in LMICs.

Our findings are consistent with prior studies that have revealed substantial underrepresentation of editorial staff members of major biomedical journals who are affiliated with LMICs.^9,10,11,12,19,20^ One 2005 study reported that 96% of editorial staff members of leading pediatric journals were based in HICs,^21^ and another study in 2014 reported that 93% of editorial staff members of leading orthopedics journals were based in HICs.^9^ Similarly, in 2020, we found that 95% of the cumulative total of editorial staff members in the selected journals with high impact factors across various fields were based in HICs. Our analysis additionally indicated that all editors-in-chief were affiliated with HICs. Although the leading journals included in our analysis are meant to serve as international platforms for advancing clinical science, several journals included were national or regional journals (eg, *JAMA* [the *Journal of the American Medical Association*] and the *European Heart Journal*); thus, it was expected that many editors-in-chief would be affiliated with similar regions. Notwithstanding this expectation, editorial staff members affiliated with LMICs were underrepresented across multiple specialty journals.

The question of editorial staff affiliation is crucial in defining the priorities of a journal, as well as subsequent published scientific output. The underrepresentation of editorial staff affiliated with LMICs in leading biomedical journals may limit the availability of scientists from LMICs to submit articles to these journals. Such homogeneity may foster implicit bias in which research conducted in HICs or focused on health care problems common in HICs may be given preference by an editorial board comprising members predominantly from HICs, as was the case in our analysis.^4^ A 2017 study investigating implicit bias among researchers and health care professionals across the US reported that 58% of participants associated “good research” with HICs compared with LMICs.^22^ Because editorial staff were not explicitly studied in that survey, it is unclear whether such biases extend to editorial staff. In addition, in a survey of nearly 900 senior investigators in the United States, respondents reported a greater likelihood of referring manuscripts for peer review if the research was conducted in an HIC compared with work conducted in LMICs.^23^ The prevalence of such biases and the lack of substantive representation of LMICs on the editorial staff of leading journals may hinder the progress and dissemination of scientific knowledge in these settings. Our analyses revealed that senior-level editorial staff positions were almost exclusively held by individuals from HICs and upper-middle-income countries. It is further evident that individuals from lower-middle-income countries and low-income countries were most often found in advisory or other peripheral editorial roles and thus may have less influence in decisions around journal priorities and article acceptance. Investigators from LMICs may also perceive bias against their work,^13^ which may preclude them from submitting to journals with high impact factors. However, our analysis did not evaluate which articles were submitted to journals, rendering us unable to assess whether there is an underrepresentation of research conducted in LMICs that is ultimately submitted to these journals with high impact factors.

Our findings indicate that, despite containing most of the world’s population (6.5 billion in 2020 [84%])^24^ and bearing most of the world’s disease burden,^1^ LMICs generated only 22% of original research published in leading international journals in 2020. Although there has been an increase in the amount of research emerging from LMICs in the past decade, it has been disproportionately surpassed by the increase in research output from HICs.^25^ There are many historical factors associated with the structural inequalities leading to disparities in research output—most notably, the lack of research infrastructure in many LMICs.^26^ In addition, the dependence of LMICs on funding from the Global North, as well as the centralization of major publication platforms in the Global North, further undermines the current capacity for publication of research conducted in these settings.^27^ The culmination of these dynamics may foster a dependency whereby LMICs rely on the presupposed expertise of HIC decision-makers to set the agenda regarding programs and funding allocated to local investigators.^28^ There is an urgent need for high-quality research in LMICs in which the local disease burden is taken into account and empowerment of local investigators is prioritized to promote long-term and dynamic means of responding to health needs.^26^ The current lack of scientific autonomy in LMICs is a particularly dire, and unsustainable, reality.^29^

Editorial bias that may affect manuscript selection reinforces power differentials that have historically excluded LMICs from meaningful involvement in global science.^30^ Although our study did demonstrate a moderate correlation between editorial staff diversity and published articles reporting work conducted in LMICs, even in cases of journals in which LMICs were comparatively well represented, editorial staff affiliated with LMICs remained disproportionately outnumbered. On average, only 5% of the editorial staff members of each journal were affiliated with LMICs, with a range of 0% to 35% of editorial staff members affiliated with LMICs for each individual journal. Journals such as the *European Heart Journal* and the *Journal of the American College of Cardiology*, which cumulatively employed a sizable portion of the net total of LMIC editors in our analysis, remain overwhelmingly skewed in terms of representation, with LMIC editors making up only 9% and 4% of their editorial staff, respectively. Hence, it is evident that more representation of LMICs on the editorial staff of major journals is an important step toward equity in global science. International publication platforms should diversify their editorial staff to be representative of the international community. Further research is needed to assess what additional structural changes may also empower scientists from LMICs and increase publications reporting high-quality research conducted in these settings.

### Limitations

Our study is subject to several limitations. First, we analyzed data only from 2020, as journals’ websites reflected only current editorial staff at the time of our search. Furthermore, the selected journals did not uniformly report past editorial staff membership. As 2020 was the height of the COVID-19 pandemic, publications in these journals may not reflect the long-standing priorities of the journals and may have led to overrepresentation or underrepresentation of original research articles from LMICs, particularly from China. We did not specifically categorize articles as COVID-19 related. Thus, we were unable to assess the extent to which the pandemic affected our findings. We did not assess the gender^31^ or race and ethnicity^32^ of editorial staff members. Further studies are needed to assess these characteristics of the editorial staff affiliated with LMICs because there may be additional gaps noted therein. We excluded case reports, which may be more common in settings in which limited research infrastructure may preclude larger studies. Last, we were unable to assess whether greater geographic diversity among editorial staff was associated with the number of articles submitted to a given journal or the proportion of articles from LMICs that are accepted to these journals.

## Conclusions

Editorial staff members in leading journals were largely composed of individuals affiliated with HICs in North America and Europe, with 95% of editorial staff affiliated with HICs. When included, editorial staff members affiliated with LMICs largely held minor editorial roles, and none were editors-in-chief. Notwithstanding their relative infrequency as editorial staff, the inclusion of more editorial staff affiliated with LMICs was moderately correlated with increases in the proportion of published articles reporting research conducted in LMICs. Further research is needed to identify structural changes that may effectively empower scientists from LMICs and increase publications reporting high-quality research conducted in the countries where most of the world’s disease burden lies.

## Notes

Supplement. **eAppendix.** Journals Representing the Leading Journals in Fields Conferring the Greatest Disease Burden That Publish Primary Research Articles **eTable 1.** Editorial Staff Affiliations in Leading Medical Journals by Country Region **eTable 2.** Study Country Region of Original Research Articles Published in Leading Medical Journals **eFigure 1.** Association Between Editorial Staff Composition and Publications From Low- and Middle-Income Countries Among Multi-Country Studies **eFigure 2.** Association Between Editorial Staff Composition and Publications From Low- and Middle-Income Countries Among Single-Country Studies **eFigure 3.** Association Between Editorial Staff Composition by Region and the Proportion of Publications From Each Region
