# Supplementary material for: Association of International Editorial Staff With Published Articles From Low- and Middle-Income Countries
Source: JAMA Netw Open. 2022 May 23;5(5):e2213269. doi: 10.1001/jamanetworkopen.2022.13269 (PMC9127552; doi:10.1001/jamanetworkopen.2022.13269)
Supplement: Supplement. — eAppendix. Journals Representing the Leading Journals in Fields Conferring the Greatest Disease Burden That Publish Primary Research Articles eTable 1. Editorial Staff Affiliations in Leading Medical Journals by Country Region eTable 2. Study Country Region of Original Research Articles Published in Leading Medical Journals eFigure 1. Association Between Editorial Staff Composition and Publications From Low- and Middle-Income Countries Among Multi-Country Studies eFigure 2. Association Between Editorial Staff Composition and Publications From Low- and Middle-Income Countries Among Single-Country Studies eFigure 3. Association Between Editorial Staff Composition by Region and the Proportion of Publications From Each Region [file jamanetwopen-e2213269-s001.pdf]

Supplementary Online Content

Melhem G, Rees CA, Sunguya BF, Ali M, Kurpad A, Duggan CP. Association of international editorial staff with published articles from low- and middle-income countries. *JAMA Netw Open.* 2022;5(5):e2213269. doi:10.1001/jamanetworkopen.2022.13269

eAppendix. Journals Representing the Leading Journals in Fields Conferring the Greatest Disease Burden That Publish Primary Research Articles
eTable 1. Editorial Staff Affiliations in Leading Medical Journals by Country Region
eTable 2. Study Country Region of Original Research Articles Published in Leading Medical Journals
eFigure 1. Association Between Editorial Staff Composition and Publications From Low- and Middle-Income Countries Among Multi-Country Studies
eFigure 2. Association Between Editorial Staff Composition and Publications From Low- and Middle-Income Countries Among Single-Country Studies
eFigure 3. Association Between Editorial Staff Composition by Region and the Proportion of Publications From Each Region

This supplementary material has been provided by the authors to give readers additional information about their work.

© 2022 Melhem G et al. *JAMA Network Open.*

eAppendix. Journals Representing the Leading Journals in Fields Conferring the Greatest Disease Burden That Publish Primary Research Articles

**Journal**	**Impact Factor**
General Medicine	
New England Journal of Medicine	74.699
Lancet	60.390
JAMA	45.540
BMJ	30.313
Annals of Internal Medicine	21.317
Surgery	
JAMA Surgery	13.625
Annals of Surgery	10.130
Journal of Neurology Neurosurgery and Psychiatry	8.263
Journal of Heart and Lung Transplantation	7.865
Endoscopy	7.341
Pediatrics	
JAMA Pediatrics	13.946
Lancet Child & Adolescent Health	8.543
Journal of the American Academy of Child and Adolescent Psychiatry	6.936
Archives of Diseases in Childhood-Fetal and Neonatal Edition	5.436
Pediatrics	5.359
Obstetrics and Gynecology	
American Journal of Obstetrics and Gynecology	6.502
Fertility and Sterility	6.312
Human Reproduction	5.733
Ultrasound in Obstetrics and Gynecology	5.571
Obstetrics and Gynecology	5.524
Cancer	
CA: A Cancer Journal for Clinicians	292.278
Lancet Oncology	33.752
Journal of Clinical Oncology	32.956
Cancer Discovery	29.497
Cancer Cell	26.602
Cardiovascular Diseases	
Circulation	23.603
European Heart Journal	22.673
Journal of the American College of Cardiology	20.589
Circulation Research	14.467
JAMA Cardiology	12.794
Infectious Diseases	
Lancet Infectious Diseases	24.446
Lancet HIV	14.813
Clinical Infectious Diseases	8.313
Clinical Microbiology and Infection	7.117
Journal of Travel Medicine	7.089
Psychiatry	
World Psychiatry	40.595
JAMA Psychiatry	17.471
Lancet Psychiatry	16.209
Psychotherapy and Psychosomatics	14.864
American Journal of Psychiatry	14.119
Nutrition	
American Journal of Clinical Nutrition	6.766

International Journal of Behavioral Nutrition and Physical Activity	6.714
Clinical Nutrition	6.360
Journal of the International Society of Sports Nutrition	5.068
European Journal of Nutrition	4.664

© 2022 Melhem G et al. *JAMA Network Open.*

eTable 1. Editorial Staff Affiliations in Leading Medical Journals by Country Region

		Editorial Staff Country Affiliation Region						
	**Total, n**	**East Asia and Pacific, n (%)**	**Europe and Central Asia, n (%)**	**Latin America and the Caribbean, n (%)**	**Middle East and North Africa, n (%)**	**North America, n (%)**	**South Asia, n (%)**	**Sub-Saharan Africa, n (%)**
General Medicine Journals	517	12 (2.3)	151 (29.2)	1 (0.2)	2 (0.4)	347 (67.1)	0 (0.0)	4 (0.8)
Surgery Journals	411	56 (13.6)	109 (26.5)	9 (2.2)	2 (0.5)	233 (56.7)	2 (0.5)	0 (0.0)
Pediatrics Journals	156	5 (3.2)	32 (20.5)	1 (0.6)	1 (0.6)	117 (75.0)	0 (0.0)	0 (0.0)
Obstetrics and Gynecology Journals	385	18 (4.7)	136 (35.3)	9 (2.3)	9 (2.3)	211 (54.8)	2 (0.5)	0 (0.0)
Cancer Journals	207	7 (3.4)	36 (17.4)	3 (1.4)	1 (0.5)	158 (76.3)	2 (1.0)	0 (0.0)
Cardiovascular Diseases Journals	1,022	56 (5.5)	368 (36.0)	12 (1.2)	12 (1.2)	566 (55.4)	5 (0.5)	3 (0.3)
Infectious Diseases Journals	429	49 (11.4)	104 (24.2)	5 (1.2)	14 (3.3)	239 (55.7)	5 (1.2)	13 (3.0)
Psychiatry Journals	192	17 (8.9)	64 (33.3)	8 (4.2)	5 (2.6)	88 (45.8)	6 (3.1)	4 (2.1)
Nutrition Journals	500	60 (12.0)	256 (51.2)	7 (1.4)	11 (2.2)	161 (32.2)	2 (0.4)	3 (0.6)
**Total**	3,819	280	1,256	55	57	2,120	24	27

© 2022 Melhem G et al. *JAMA Network Open.*

eTable 2. Study Country Region of Original Research Articles Published in Leading Medical Journals

		Article Study Country						
	**Total, n[a]**	**East Asia and Pacific, n (%)**	**Europe and Central Asia, n (%)**	**Latin America and the Caribbean, n (%)**	**Middle East and North Africa, n (%)**	**North America, n (%)**	**South Asia, n (%)**	**Sub-Saharan Africa, n (%)**
General Medical Journals	1,323	237 (17.9%)	349 (26.4%)	96 (7.3%)	60 (4.5%)	491 (37.1%)	37 (2.8%)	53 (4%)
Surgery Journals	1,267	213 (16.8%)	420 (33.1%)	19 (1.5%)	12 (0.9%)	581 (45.9%)	15 (1.2%)	7 (0.6%)
Pediatrics Journals	815	84 (10.3%)	181 (22.2%)	19 (2.3%)	9 (1.1%)	488 (59.9%)	20 (2.5%)	14 (1.7%)
Obstetrics and Gynecology Journals	1,293	248 (19.2%)	407 (31.5%)	31 (2.4%)	34 (2.6%)	546 (42.2%)	14 (1.1%)	13 (1%)
Cancer Journals	879	174 (19.8%)	224 (25.5%)	34 (3.9%)	33 (3.8%)	404 (46%)	5 (0.6%)	5 (0.6%)
Cardiovascular Diseases Journals	1,382	220 (15.9%)	432 (31.3%)	57 (4.1%)	47 (3.4%)	543 (39.3%)	46 (3.3%)	37 (2.7%)
Infectious Diseases Journals	2201	479 (21.8%)	539 (24.5%)	128 (5.8%)	57 (2.6%)	665 (30.2%)	86 (3.9%)	247 (11.2%)
Psychiatry Journals	268	26 (9.7%)	101 (37.7%)	10 (3.7%)	6 (2.2%)	118 (44%)	5 (1.9%)	2 (0.7%)
Nutrition Journals	1,600	440 (27.5%)	691 (43.2%)	106 (6.6%)	64 (4%)	252 (15.8%)	21 (1.3%)	26 (1.6%)

[a]There were 543 articles published that reported work that was conducted in countries from >1 geographical region.

© 2022 Melhem G et al. *JAMA Network Open.*

eFigure 1. Association Between Editorial Staff Composition and Publications From Low- and Middle-Income Countries Among Multi-Country Studies

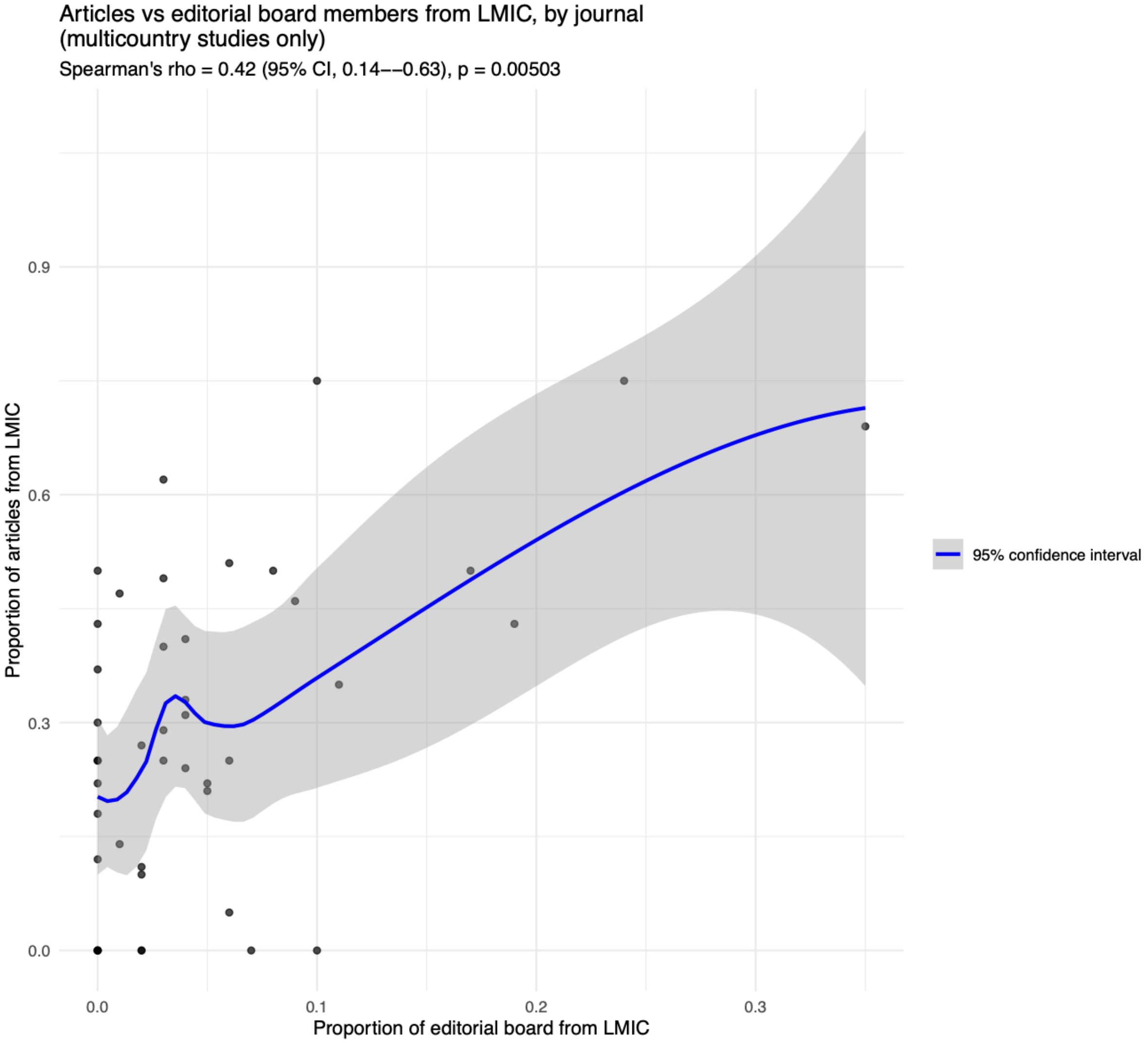

© 2022 Melhem G et al. *JAMA Network Open.*

eFigure 2. Association Between Editorial Staff Composition and Publications From Low- and Middle-Income Countries Among Single-Country Studies

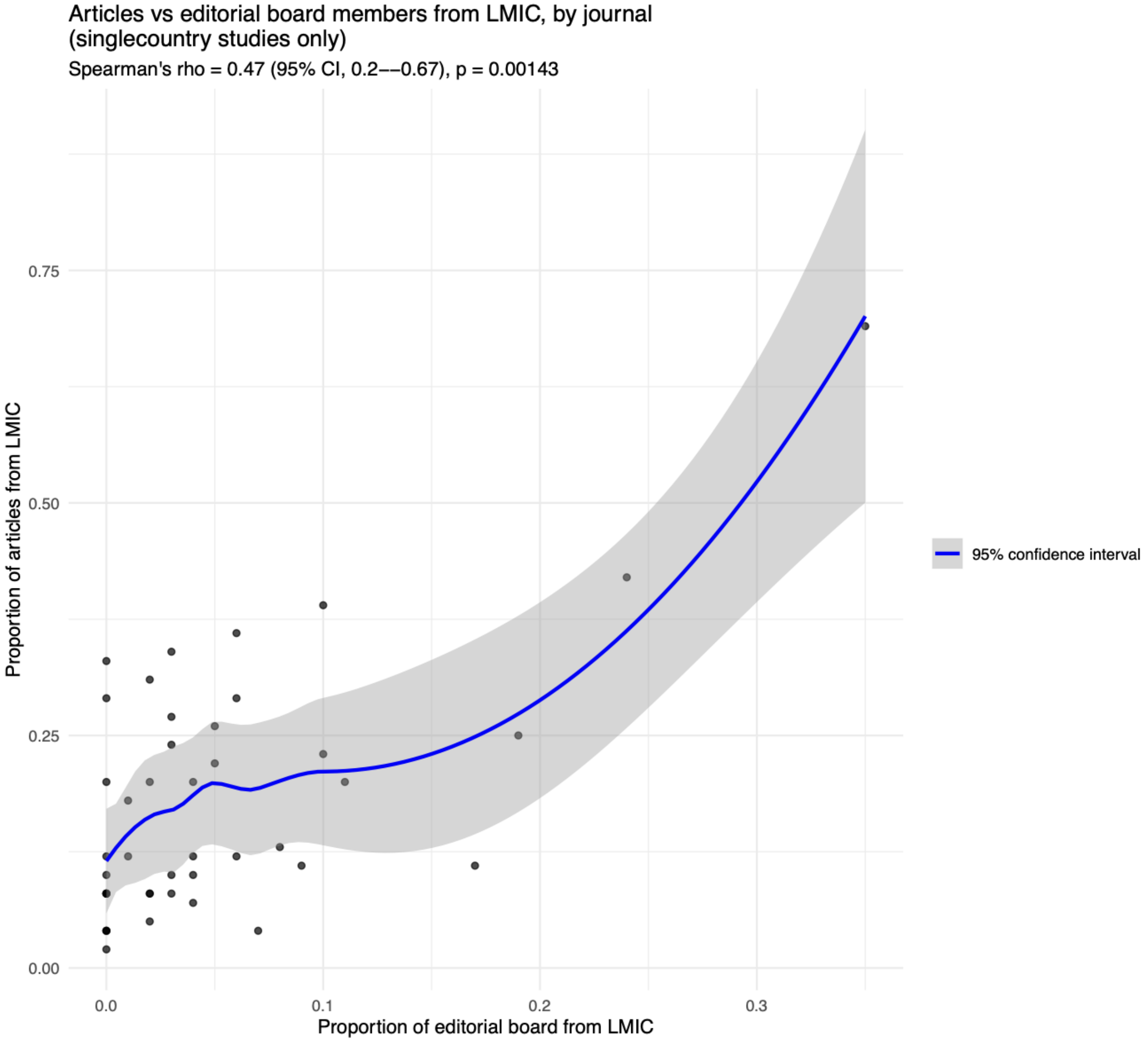

© 2022 Melhem G et al. *JAMA Network Open.*

eFigure 3. Association Between Editorial Staff Composition by Region and the Proportion of Publications From Each Region

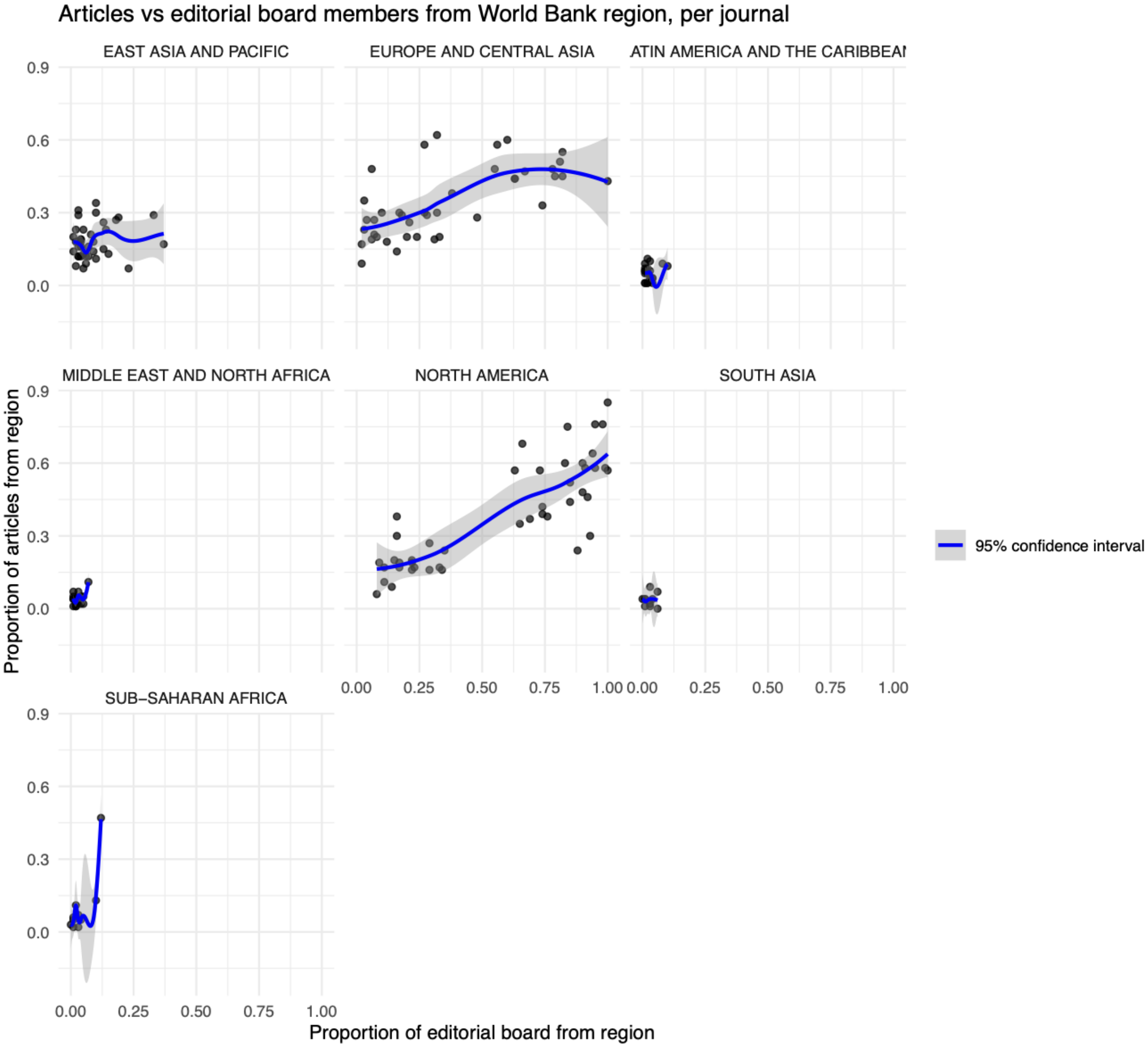

© 2022 Melhem G et al. *JAMA Network Open.*
